# Osteoporosis: a cardiovascular risk factor equivalent to type 2 diabetes

**Published:** 2010

## Introduction

An increasing body of research is showing a link between osteoporosis and cardiovascular disease. The associations are complex and, in some cases, not yet fully understood, but the clinical implications are that healthcare professionals need to consider their osteoporosis patients as having as high a risk for heart disease as those with type 2 diabetes mellitus.

Prof Erik Eriksen, professor of Endocrinology and Internal Medicine at Oslo University Hospital, Aker, Norway, explains further. He was in South Africa recently at the invitation of Novartis. ‘Bone and blood vessels are closely linked’, he told local general practitioners and specialists. ‘To put it simplistically, as the skeleton loses calcium, that calcium builds up in the arterial walls. This phenomenon is what spurred the interest in the relationship and led to research initiatives.’

Bone remodelling – the process whereby the skeleton constantly repairs itself – is a dynamic process that becomes accelerated in osteoporosis, leading to a negative bone balance. The remodelling process leads to the release of a variety of substances (such as cytokines and growth factors) linking bone to blood vessels. These are potentially harmful in accelerated states like osteoporosis. Prof Eriksen quoted some startling findings from the MORE study undertaken by Tanka et al. in 2005:

Osteoporotic women have a 3.9-fold increased risk of cardiovascular events relative to women with osteopenia.
Each one standard deviation decrease in bone-mineral density is associated with a 70% increase in stroke risk.
Aortic calcification is an independent predictor of bone-mineral density (BMD).
There is a graded relationship between bone loss and progression to vascular calcification.
There is a significant relationship between risk of myocardial infarction (MI) and low BMD.

The osteoprotegerin (OPG) and rank ligand (RANKL) system is thought to play a role in the relationship between osteoporosis and cardiovascular disease. RANKL has been implicated in potentially catastrophic plaque rupture that can lead to stroke or MI. ‘However, there are currently some conflicting results in respect of OPG, based on mouse models’, said Prof Eriksen.

CTX-1, a bone marker, has also been shown to have cardiovascular effects. Prof Eriksen cited an Australian study in which higher levels of CTX-1 were associated with higher cardiovascular risk as well as increased mortality. Conversely, vitamin K would appear to have positive effects on bone matrix proteins, so high levels of vitamin K should be maintained.

There is increasing evidence that vascular cells acquire osteoblast characteristics and that arterial cells can differentiate into osteoclasts. ‘This means that when we treat high bone turnover with anti-resorptive drugs, we also help to treat cardiovascular disease’, said Prof Eriksen. ‘Although we’re still not sure why, we have found that zoledronic acid 5 mg reduces the risk of all-cause mortality over time. A new study has shown that use of bisphosphonate is significantly associated with reduced mortality in the first three years after hip fracture, very possibly a double effect in that it may also be protecting against cardiovascular disease.’

In summary, Prof Eriksen reiterated that, based on a coherent array of results from animal studies and in vitro experiments, numerous factors have been shown to affect bone and vasculature and that osteoporotic patients are at increased cardiovascular risk as a result of their accelerated bone turnover. ‘Decarboxylation of matrix proteins consequent on vitamin K deficiency and high bone turnover may constitute an important pathogenetic pathway. Treatment with bisphosphonates may therefore be of benefit’, he concluded.

